# Differences in the impact of the largest dengue epidemic outbreak in Peru’s history and lessons learned

**DOI:** 10.17843/rpmesp.2023.404.13151

**Published:** 2023-12-18

**Authors:** Martín Casapía-Morales, Juan C. Celis-Salinas, Stalin Vilcarromero, Miguel Villegas-Chiroque, Alejandro Llanos-Cuentas

**Affiliations:** 1 School of Medicine, National University of the Peruvian Amazon, Iquitos, Peru National University of the Peruvian Amazon School of Medicine National University of the Peruvian Amazon Iquitos Peru; 2 Department of Infectious and Tropical Diseases, “Felipe Arriola Iglesias” Regional Hospital of Loreto, Iquitos, Peru. Department of Infectious and Tropical Diseases “Felipe Arriola Iglesias” Regional Hospital of Loreto Iquitos Peru; 3 Infectious Diseases Department, Edgardo Rebagliati Martins National Hospital, Buenos Aires, ESSALUD, Lima, Peru. Infectious Diseases Department Edgardo Rebagliati Martins National Hospital, ESSALUD Lima Peru; 4 Infectious Diseases Service, Lambayeque Regional Hospital, Chiclayo, Peru. Infectious Diseases Service Lambayeque Regional Hospital Chiclayo Peru; 5 Alexander Von Humboldt Institute of Tropical Medicine, Peruvian University Cayetano Heredia, Lima, Peru. Peruvian University Cayetano Heredia Alexander Von Humboldt Institute of Tropical Medicine Peruvian University Cayetano Heredia Lima Peru

To the editor. From January to July 2023, Peru faced the largest dengue outbreak in its history with more than 192,382 cases, 8599 hospitalizations and 249 confirmed deaths ^1^. This caused the collapse of health services in several cities because there were not enough beds for the large number of patients. Nevertheless, there was a notable difference between the response of the services, mortality and complications of the patients among the regions of our country ^2^.

In order to determine the impact of this epidemic dengue outbreak in two selected regions, epidemiological data and hospital indicators were analyzed for Lambayeque, a region with no experience in dealing with large outbreaks, and compared with Loreto, a region that has had multiple outbreaks, including some very large and severe ones over the last 30 years. The hospital and regional data are freely available, and the information that was not available was requested from the Centers for Disease Prevention and Control and Metaxenic Disease Strategies. In order to provide recommendations and identify the lessons learned to help control the impact of this outbreak, technical meetings were held with seven experts, including infectious disease specialists and epidemiologists, from the Lambayeque and Loreto regions. In addition, six key informants participated, including managers and those responsible for the strategies in the regions. This initiative was presented to the regional health authority of Loreto and Lambayeque in the context of the epidemic (supplementary figure).

During the study period, 23,527 cases were reported in Lambayeque, with 86 deaths ^2^; only one death was reported in Loreto. The case fatality rate of the outbreak was 40 times higher in Lambayeque than in the Loreto region (supplementary table). The case fatality rate for severe dengue in Lambayeque and Loreto was 86.9% (93/107) and 9.0% (1/11), respectively. The epidemiological notification data system has some limitations such as: the final definition of probable cases who did not have laboratory confirmation, incomplete data, and the fact that the quality of the information depended on the person who made the report, therefore, there is a trend towards underreporting in the epidemiological notification system, which may increase in times of health emergencies ^3^. Furthermore, the data provided by different offices may be discordant. Finally, after contrasting the epidemiological data from Loreto and Lambayeque, we present the lessons learned according to key informants and experts are presented in Table 1 ^4^.

**Table 1:** Lessons learned. Dengue outbreak in Peru, 2023.

1.	To design a realistic plan in advance, taking into account the maximum bed capacity in available areas.
2.	The plan should include training in the correct management of dengue according to national guidelines, before and during the outbreak ^4^.
3.	I-4 health centers, as a first barrier, should be prepared to hospitalize patients with dengue fever and warning signs.
4.	Intensive communication campaign aimed at identifying warning signs.
5	Implementation of the plan requires budget and political support, including adequate vector control and trained human resources.
6.	Each region should have a plan according to its local characteristics.
7.	Support from professional experts from other regions.
8.	Consider including infectious disease specialists in hospitals in affected areas.
9.	Seroprevalence studies and confirmation of the diagnoses of reported cases are needed in order to improve decision making.

In conclusion, it is critical that government authorities and policy makers consider the experience of personnel from endemic areas regarding the management and control of dengue outbreaks and thus prepare for the next epidemics. The threat is imminent in view of climate change, such as a new El Niño Global phenomenon, which, together with insufficient vector control and other socioeconomic conditions, favors the appearance of epidemic outbreaks ^5^ of dengue and other arboviruses such as Zika and chikungunya, even in non-endemic regions. Consequently, it is imperative to understand and act on what has been learned so that avoidable deaths do not continue to occur.
