# Supplementary material for: Differences in the impact of the largest dengue epidemic outbreak in Peru’s history and lessons learned
Source: Rev Peru Med Exp Salud Publica. 2023 Dec 18;40(4):493–4. doi: 10.17843/rpmesp.2023.404.13151 (PMC11138827; doi:10.17843/rpmesp.2023.404.13151)
Supplement: Supplementary material. — Available in the electronic version of the RPMESP. [file rpmesp-40-04-13151-s001.pdf]

Tabla suplementaria. Indicadores demográficos y hospitalarios de dengue en Loreto y Lambayeque, Perú. Enero-mayo 2023.

**Regiones**	**Loreto**	**Lambayeque**
Casos de dengue	8,948	23,527
Hombres (%)	46.7	43.4
< 11 años (%)	30.1	14.2
18 a 60 años (%)	46.5	64.9
Casos con signos alarma (%)	7776(86.9)	2989(88.4)
Dengue sin signos de alarma (%)	1154(12.9)	17397(11.0)
Incidencia/100,000 habitante	847.0	1734.0
Fallecidos	1	93
Letalidad	0.01	0.40
Epidemias anteriores	Si	No
Hospitalizados	354	159
Dengue grave	11	107
Serotipos circulantes previamente	1,2,3,4	1,2
Serotipos circulantes actualmente	1,2(cosmopolita)	1,2(cosmopolita)

Figura 1. Integrantes y flujo de las reuniones técnicas de expertos e informantes claves que trabajan en el control de dengue en Lambayeque y Loreto, 2023

REUNIÓN N° 1
Tema: Control de Brote epidémico dengue 2023

Región Loreto, ciudad Iquitos
Convocatoria:
Gerencia Regional de Salud Loreto

Integrantes:
- Infectólogos del Hospital Regional de Loreto [5]
- Director de centro y control de enfermedades de la GERESA Loreto [1]
- Epidemiólogo de la GERESA Loreto [1]
- Director y coordinador de control vectorial de enfermedades metaxénicas [2]

→

REUNIÓN N° 2
Tema: Control de Brote epidémico dengue 2023
Lugar:
Región Lambayeque, ciudad Chiclayo
Convocatoria:
Gerencia Regional de Salud Lambayeque

Integrantes:
- Infectólogo del Hospital Regional de Lambayeque, MINSA [1] y cardiólogo del Hospital Almanzor Aguinaga Asenjo Chiclayo, ESSALUD [1]
- Gerente Regional de Salud [GERESA] de Lambayeque [1]
- Coordinador regional de prevención y control de enfermedades metaxénicas[1]
- **Experto invitado de Loreto (socializar experiencia) [1]**

Agenda:
- Sala situacional actual de dengue en la región
- Manejo de los casos de los casos de dengue ambulatorio y hospitalario
- Mejor forma de lograr la prevención de dengue y participación de la comunidad
- Control vectorial efectivo
- Capacitación del personal clínico y de control vectorial por personal experto
- Lecciones aprendidas

METODOLOGÍA Y PERMISOS:
- La reunión técnica en Loreto, fue convocada, como es usual, debido al incremento de casos de dengue y fue convocado por la GERESA Loreto, conformando al grupo de expertos disponibles (ver figura), pero además, respondía el pedido de apoyar a la GERESA Lambayeque en el contexto del brote epidémico de Dengue. El experto de dengue elegido de Loreto, acudió a la región Lambayeque y participo de la reunión técnica convocada por la GERESA Lambayeque para responder la Agenda señalada previamente desarrollada en Loreto, de modo que, posteriormente, sea socializada como lecciones aprendidas, y a todo nivel, para poder disminuir el impacto de futuros brotes epidémicos.
- Los datos epidemiológicos fueron de acceso publico de las GERESA y del MINSA, especialmente de: https://www.dge.gob.pe/sala-situacional-dengue/diaria/
- La tasa de letalidad fue utilizada calculando los casos fallecidos confirmados sobre los casos de dengue en general.
