# Supplementary material for: Differences in the impact of the largest dengue epidemic outbreak in Peru’s history and lessons learned
Source: Rev Peru Med Exp Salud Publica. 2023 Dec 18;40(4):493–4. doi: 10.17843/rpmesp.2023.404.13151 (PMC11138827; doi:10.17843/rpmesp.2023.404.13151)
Supplement: Translation

# Translation: Diferencias en el impacto del mayor brote epidémico de dengue en la historia del Perú y lecciones aprendidas

**Authors:** Martín Casapía-Morales, Juan C. Celis-Salinas, Stalin Vilcarromero, Miguel Villegas-Chiroque, Alejandro Llanos-Cuentas

**Affiliations:** 1 Facultad de Medicina, Universidad Nacional de la Amazonía Peruana, Iquitos, Perú.; 2 Departamento de Enfermedades Infecciosas y Tropicales, Hospital Regional de Loreto «Felipe Arriola Iglesias», Iquitos, Perú.; 3 Servicio de Infectologia, Hospital Nacional Edgardo Rebagliati Martins, ESSALUD, Lima, Perú.; 4 Servicio de Infectología, Hospital Regional Lambayeque, Chiclayo, Perú.; 5 Instituto de Medicina Tropical Alexander Von Humboldt, Universidad Peruana Cayetano Heredia, Lima, Perú.

Sr. Editor. Desde enero a julio del 2023, el Perú enfrentó el brote más grande de dengue en toda su historia con más de 192 382 casos, 8599 hospitalizaciones y 249 fallecidos confirmados ^(^^1^. Esto ocasionó el colapso de los servicios de salud en varias ciudades porque no había suficientes camas para el gran número de pacientes. No obstante, se presentó una notable diferencia entre la respuesta de los servicios, mortalidad y complicaciones de los pacientes entre las regiones de nuestro país ^(^^2^.

Con el objetivo de determinar el impacto de este brote epidémico de dengue en dos regiones seleccionadas, se analizaron datos epidemiológicos e indicadores hospitalarios de Lambayeque, región sin experiencia afrontando grandes brotes; y se les comparo con Loreto, región que ha tenido múltiples brotes, incluido algunos muy grandes y severos en los últimos 30 años. Estos datos hospitalarios y regionales eran de libre acceso, y los que no estaban disponibles, fueron solicitados a los centros de prevención y control de enfermedades y las estrategias de enfermedades metaxénicas. Asimismo, con la finalidad de poder establecer algunas recomendaciones e identificar las lecciones aprendidas que ayuden al control de este impacto, se realizaron reuniones técnicas con siete expertos, entre infectólogos y epidemiólogos, de la región Lambayeque y Loreto. Adicionalmente participaron seis informantes claves que incluían gerentes y responsables de las estrategias en las regiones. Esta iniciativa fue presentada a la autoridad regional de salud de Loreto y Lambayeque en el contexto de la epidemia (figura suplementaria).

Durante este periodo, en Lambayeque se habían registrado 23 527 casos, con 86 fallecidos ^2^, mientras que en Loreto se había reportado un fallecido. La tasa de letalidad del brote fue 40 veces más alta en Lambayeque respecto a la región Loreto (tabla suplementaria). Y la tasa de letalidad por dengue severo en Lambayeque y Loreto fue de 86.9% (93/107) y 9.0% (1/11), respectivamente. Debe mencionarse que existen limitaciones en los datos de la notificación epidemiológica tales como: la definición final de los casos probables que no tienen confirmación laboratorial; la presencia de datos incompletos, la dependencia de la calidad de la información por quien realiza el reporte, y por lo tanto, una tendencia hacia a un subregistro en el sistema de notificación epidemiológica, y es posible que esto sea mayor en tiempo de emergencias sanitarias^3^. Y, además, los datos proporcionados por las diferentes oficinas pueden ser discordantes. Finalmente, luego de contrastar los datos epidemiológicos de Loreto y Lambayeque, las lecciones aprendidas según los informantes claves y expertos se presentan en la tabla 1 ^4^.

**Tabla 1:** Lecciones aprendidas. Brote de dengue en Perú, 2023.

1. Diseñar un plan realista anticipadamente, que considere la capacidad máxima de camas en áreas disponibles.
2. El plan debe incluir capacitación en correcto manejo de dengue de acuerdo a guía nacional, antes y durante el brote (4).
3. Centros de salud I-4 como primera barrera de contención deben acondicionarse para hospitalizar pacientes con dengue y signos de alarma.
4. Campaña comunicacional intensa dirigida a la identificación de signos de alarma.
5 Ejecución del plan requiere presupuesto y apoyo político, debiendo incluirse el control vectorial adecuado y con recursos humanos capacitados.
6. Cada región debe tener plan de acuerdo con sus características locales.
7. Apoyo de profesionales expertos de otras regiones.
8. Considerar incluir especialistas en enfermedades infecciosas en hospitales de áreas afectadas.
9. Es necesario realizar estudios de seroprevalencia y confirmar los diagnósticos de los casos notificados para mejorar la toma de decisiones.

En conclusión, es crítico que las autoridades de los gobiernos y decisores políticos consideren la experiencia en el manejo y el control de brotes de dengue del personal de las areas endémicas y con ello se preparen para las próximas epidemias, cuya amenaza es inminente teniendo en cuenta el cambio climático expresado como un nuevo fenómeno de El Niño Global que, sumado al insuficiente control vectorial y otras condiciones socioeconómicas favorecen la aparición de brotes epidémicos ^5^ de dengue y otras arbovirosis como zika y chikungunya, incluso en regiones no endémicas. En consecuencia, es perentorio que se comprenda y actúe en función de lo aprendido para que no sigan presentándose muertes que pueden ser evitadas.
